# Attachment style and post-bariatric surgery health behaviours: the mediating role of self-esteem and health self-efficacy

**DOI:** 10.1186/s40359-023-01273-5

**Published:** 2023-08-25

**Authors:** Johanna Eveliina Pyykkö, Chris Hinnen, Ömrüm Aydin, Max Nieuwdorp, L. Maurits De Brauw, Sjoerd C. Bruin, Nienke van Olst, Victor E. A. Gerdes, Robbert Sanderman, Mariët Hagedoorn

**Affiliations:** 1 Department of Health Psychology, Faculty of Medical Sciences, University of Groningen, University Medical Center Groningen, Antonius Deusinglaan 1, Groningen, 9713 AV The Netherlands; 2 LUMC Oncology Centre, Leiden University Medical Centre, Leiden, The Netherlands; 3 Department of Internal Medicine, Spaarne Gasthuis, Hoofddorp, The Netherlands; 4 Department of Vascular Medicine, Amsterdam UMC, Amsterdam, The Netherlands; 5 Department of Metabolic and Bariatric Surgery, Spaarne Gasthuis, Hoofddorp, The Netherlands

**Keywords:** Attachment style, Self-esteem, Self-efficacy, Diet adherence, Exercise, Bariatric surgery

## Abstract

**Background:**

Attachment avoidance and anxiety have been linked to overweight and poor health behaviours, yet the mechanisms that underpin the relationship between attachment and health behaviours are not fully understood. Self-esteem and self-efficacy have been found to differ between attachment styles, rendering these variables potential mediators of the relationship. This longitudinal study investigated the serial mediation between preoperative attachment and 2-year post-operative health behaviours through self-esteem and health self-efficacy.

**Methods:**

Participants were 263 bariatric surgery patients (75.7% females, aged 47.7 ± 10.4 years, BMI 38.9 ± 3.6 kg/m^2^) assessed before the operation and again one and two years after the surgery. Patients completed the Experiences for Close Relationships Brief Scale, Rosenberg Self-esteem scale, Weight Efficacy Lifestyle Questionnaire, Bariatric Surgery Self-Management Questionnaire, Exercise Self-Efficacy Scale and the Exercise Behaviour Scale.

**Results:**

Higher preoperative attachment anxiety and avoidance were associated with lower self-esteem one year after bariatric surgery and poorer health self-efficacy two years after the surgery. Self-esteem and health self-efficacy mediated the relationships between preoperative anxious and avoidant attachment and 2- year post-operative diet adherence and physical activity.

**Conclusions:**

Helping patients to feel more worthy and reinforcing their beliefs about their own competences could lead to higher engagement with healthy lifestyle and adherence to treatment protocols, ultimately helping patients to achieve their goals for bariatric surgery.

**Clinical trial registration:**

BARIA: Netherlands Trial Register: NL5837 (NTR5992) https://www.trialregister.nl/trial/5837. Diabaria: ClinicalTrials.gov identifier (NCT number): NCT03330756.

**Supplementary Information:**

The online version contains supplementary material available at 10.1186/s40359-023-01273-5.

## Introduction

Patients’ ability to adopt and maintain a healthy lifestyle, including regular physical exercise and a healthy diet, is paramount to reaching and maintaining weight loss and optimal health outcomes long after bariatric surgery [1–3]. Promoting patients’ healthy lifestyle after bariatric surgery remains challenging, as psychological characteristics linked to better surgical outcomes and the mechanisms between patient characteristics and health behaviours are not well understood [4, 5]. A better understanding of factors related to patients’ competence to follow a healthy lifestyle after bariatric surgery could contribute to improving patient care and surgical outcomes.

Attachment style impacts health through its influence on health behaviours, symptom perception, amplification and reporting, healthcare use, stress response, emotion regulation, and access to social support [6–10]. Individual differences in attachment style reflect the beliefs and expectations people have formed in childhood about the behaviour, availability and responsiveness of the self and others in inter-personal relationships [11]. Attachment style is conceptualised as varying between attachment *anxiety* and *avoidance*. Attachment anxiety reflects a fear of abandonment and hyper-activation of attachment-seeking strategies (characterised by desperate attempts to elicit support from others by exaggerating symptoms and distress), while attachment *avoidance* manifests as a fear of intimacy and deactivating attachment strategies (e.g., ignoring, suppressing or denying emotions and symptoms to remain independent) [12–16]. Attachment anxiety and avoidance are linked to more unhealthy eating behaviours, including binge and emotional eating [17–19], which, in turn, are associated with poor weight management and poorer weight results after bariatric surgery [20–23]. Certain pathways underlying attachment and health behaviours have been widely reported before, for example, through stress response and emotion regulation [e.g., 7,9]. More specifically, anxious and avoidant attachment styles have been suggested to contribute negatively to health by influencing individuals’ physiological stress responses (e.g. increased perceived stress, prolonged intensity and duration of stress response), use of external emotion regulation strategies by relying on external substances (e.g. smoking, alcohol and high-calorie food), misuse of health services, and risky health behaviours [7, 8]. Emotion regulation has been found to mediate the relation between avoidant and anxious attachment styles and emotional eating, uncontrolled eating and binge eating among bariatric surgery patients [19, 24]. However, alternative pathways, for example, through self-esteem and self-efficacy, remain poorly studied.

Self-esteem and self-efficacy are promising mediators of the relationship between attachment style and health behaviours, given previous findings identifying differences in self-esteem and self-efficacy between different attachment styles. Furthermore, both variables are malleable traits, rendering them useful for clinical practice [25–27]. Defined as an individual’s general positive or negative evaluation of or attitude toward the self [28], self-esteem is associated with positive health practices, including regular exercise, healthier eating, better sleep behaviour, and lower eating pathology and substance use, as well as fewer physical and mental health complaints [29–31]. Attachment theory postulates that both self-esteem and attachment style stems from the experienced interactions with others, which consequently lead individuals to develop positive or negative models of the self and others [12, 32–35]. From a theoretical perspective, individuals with an anxious attachment style have formed a negative self-model, while avoidantly attached individuals perceive the self positively [12, 33]. However, increasing evidence shows that people with higher attachment anxiety and avoidance tend to report lower self-esteem [e.g., 36–39]. Individuals low on both attachment anxiety and avoidance have developed a positive model of the self and others, and show high self-esteem [12, 33, 40].

In addition to self-esteem, individuals’ belief in their ability to achieve a particular goal or outcome (i.e., self-efficacy) is an important predictor of the actual behaviour [41, 42]. Self-esteem could impact self-efficacy as our beliefs about ourselves influence the tasks we choose and the level of effort and persistence we use [43]. Existing research has established the pivotal role of self-efficacy in health behaviour change and maintenance. Self-efficacy has been found to predict various health behaviours, including weight control, exercise and nutritional intake [44–47]. The negative self-model held by more anxiously attached individuals may also explain their lower self-efficacy. In contrast, highly avoidant individuals with their positive self-image avoid seeing personal weaknesses, and may thus present higher self-efficacy [48, 49]. However, the empirical evidence for this relationship is inconclusive as attachment anxiety and avoidance were found to predict poorer self-efficacy among some populations [50–53] yet not among others [54].

### The Present Research

**Fig. 1:**
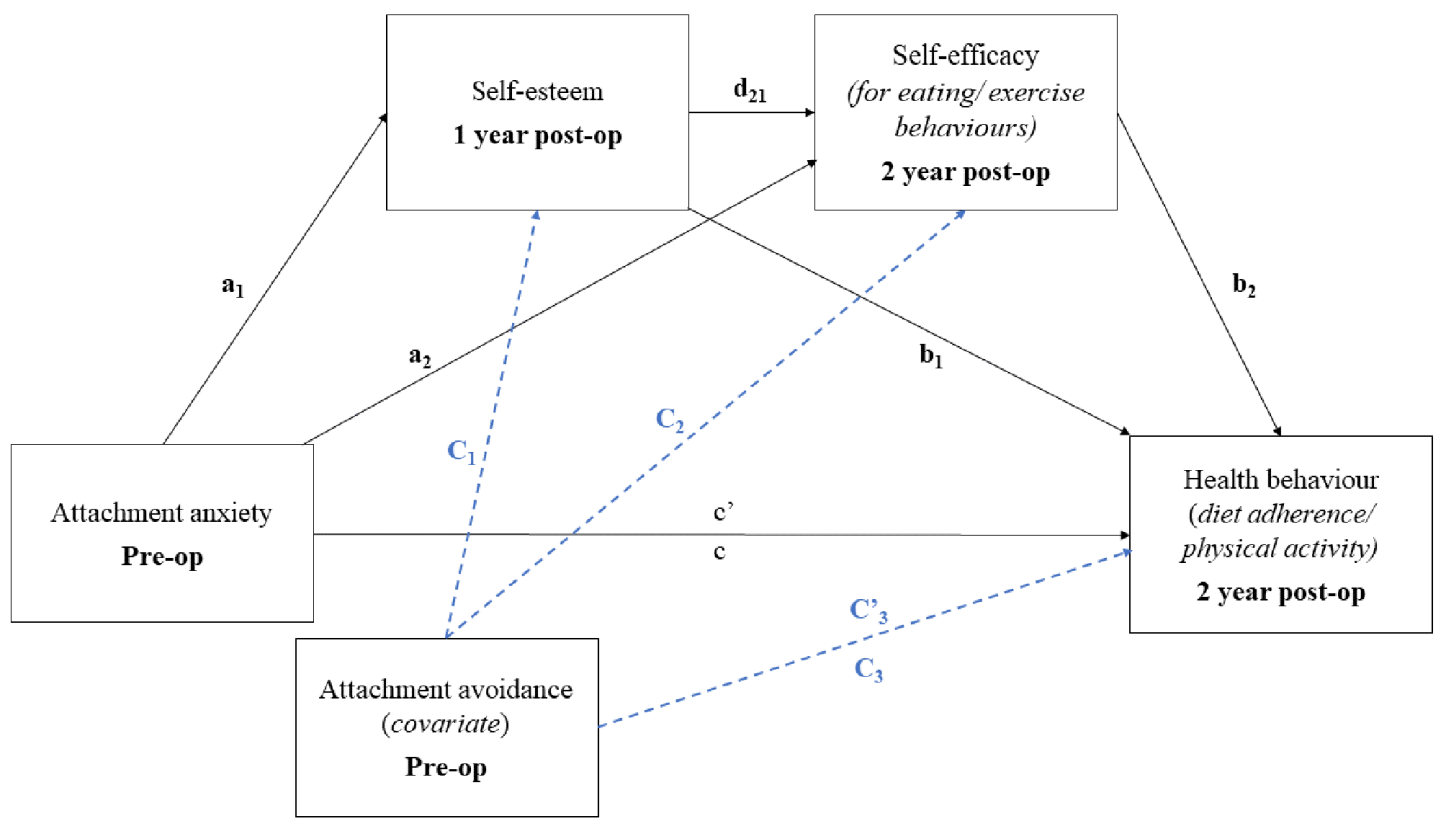
Serial multiple mediation model of attachment anxiety on health behaviour, as mediated by self-esteem and self-efficacy for a specific health behaviour, while controlling for attachment avoidance. Model 1–2: Y = Diet adherence, Model 3–4: Y = Physical activity

The studies reviewed above remain narrow in focus dealing only with individual relations between the variables, and have relied mostly on cross-sectional data. Based on attachment theory and empirical literature on bivariate associations between attachment, self-esteem and self-efficacy are posited as serial mediators in the association between attachment anxiety and avoidance and health behaviours (i.e., diet adherence and physical activity) in bariatric surgery patients. To study this longitudinally, we included preoperative attachment anxiety and avoidance as predictors, 1-year post-operative self-esteem and 2-year post-operative self-efficacy for eating and exercise behaviours as mediators, and dietary adherence and physical activity two years after bariatric surgery as the outcome variables. The conceptual model is depicted in Fig. 1.

Based on recent research findings, we expected higher preoperative attachment anxiety to be associated with poorer post-operative self-esteem and health self-efficacy. As the literature is less clear about the associations between attachment avoidance and self-esteem and health self-efficacy, we will explore these relationships. Secondly, we hypothesised higher preoperative attachment anxiety and avoidance to be associated with poorer 2- year post-operative dietary adherence and less physical activity. Lastly, we hypothesised that post-operative self-esteem and health self-efficacy would be a mediator for the relationship between attachment anxiety and dietary adherence and physical activity. These mediating pathways will also be explored for attachment avoidance. This study is the first to assess these factors in a longitudinal setting among patients with severe obesity and could provide valuable insight and recommendations for aiding patients in adhering to healthier lifestyles, ultimately leading to stable health outcomes following bariatric surgery.

## Method

### Design

The current study employed a longitudinal design and was embedded within two ongoing research projects, namely the BARIA and Diabar- studies. Patients were recruited from the outpatient clinics of Surgery and Internal medicine at MC Slotervaart (Amsterdam), Spaarne Gasthuis (Hoofddorp) and Franciscus Gasthuis (Rotterdam) in the Netherlands. More details of the BARIA-study are described in a protocol paper [55]. The Diabar- project follows the same design and procedures as the BARIA study, with the exception that only patients with diabetes are eligible to participate [56]. Both studies were approved by the Medical Research Ethics Committee of the Academic Medical Center Amsterdam (approval codes: NL55755.018.15 and NL61882.048.17) prior to commencing the study, and conducted in accordance with the Declaration of Helsinki and the Medical Research Involving Human Subjects Act (WMO).

### Participants

Participants had to have a BMI over 35 kg/m^2^ with obesity-related comorbidity or a BMI above 40 kg/m^2^; be eligible for bariatric surgery and recruited from an experienced bariatric surgery clinic; aged over 18 years; and able to provide informed consent to be included in the study. Written informed consent was obtained from all patients included in the study. Participants of the Diabar (*n* = 32) had also type 2 diabetes and the need for antidiabetic medication as inclusion criterion. We used data collected until March 31st, 2022 for the current analyses. By then, 586 patients had completed the preoperative assessment point, of these 492 had undergone surgery, and 264 had completed both 1- and 2- year post-operative assessments. Thus, we excluded in total 322 patients from the analyses for the following reasons: 47 were not operated on yet, 147 patients had not reached the follow-up assessments and 128 were lost to follow-up. The latter group includes 35 participants who withdrew from the study, 26 who did not show up for the follow-up appointment and the rest (*n* = 67) were excluded for various reasons (e.g. pregnancy, death, or missing data). Figure A1 (additional files) shows the flow of data inclusion.

### Measures

Preoperative demographic data were collected during a clinical assessment and included gender, age, ethnicity, education, occupation, marital status, and start of obesity. A clinician measured patients’ weight at every hospital visit. We used BMI (kg/m^2^), percentage of adjustable weight loss (%AWL = ((BMI_pre-op_ - BMI_post-op_)/BMI_pre-op_-13) ×100 [57] and percentage of total weight loss (%TWL = ((BMI_pre-op_ - BMI_post-op_)/BMI_pre-op_ ×100 to describe patients’ weight and weight loss. Participants completed a survey consisting of Dutch versions of validated psychological questionnaires preoperatively and then annually at each follow-up visit.

#### Attachment style

The Experiences in Close Relationships scale (ECR-M16) [58] is a 16- item scale measuring attachment style. The attachment anxiety subscale includes eight items such as ‘I worry that others will abandon me’ and eight items for attachment avoidance subscale such as ‘I try to avoid getting too close to other people’. Responses were given on a 7-point Likert scale ranging from 1 (*disagree*) to 7 (*agree*) and scores for each subscale ranged from 8 to 56, with higher scores representing higher attachment insecurity. For this study, the internal consistency was good for the preoperative anxiety subscale (Cronbach’s α = 0.86), and acceptable for the avoidance subscale (α = 0.78).

#### Self-esteem

The widely used and well validated Dutch Rosenberg self-esteem scale [59] was used to assess person’s global evaluation of him/herself. The measure consists of 10 items answered on a Likert scale ranging from 0 *(strongly disagree)* to 3 *(strongly agree)*, that generates a total score ranging from 0 to 30, with higher scores indicating higher global self-esteem. Items include statements such as ‘On the whole, I am satisfied with myself’ and ‘I certainly feel useless at times’ (reverse-scored). The internal consistency was high in the current sample (Cronbach’s alpha was α = .90) at the 1 year- follow up assessment.

#### Self-Efficacy for Controlling Eating Behaviours

The Weight Efficacy Lifestyle Questionnaire [60]) was used to assess participants’ self-efficacy for controlling their eating behaviour in specific situations. The WEL-Q consists of 20 statements, such as ‘I can resist eating even when I am in pain’ and ‘I can control my eating on the weekends’. The scale yields 5 subscales: negative emotions, availability, social pressure, physical discomfort and positive activities, as well as a total score. The responses ranged from 0 (*not confident*) to 10 (*very confident*). Scores were recoded so the total scale ranged from 0 to 100, with 100 meaning the greatest possible self-efficacy for controlling one’s eating. The total score was used in the current study and the internal consistency for the 2-year follow up assessment was excellent (Cronbach’s α = 0.96).

#### Adherence to Dietary Recommendations

The eating behaviour subscale of the Bariatric Surgery Self-Management Questionnaire [61]) was used to assess adherence to the eating recommendations during the past week. The subscale consists of eight items, such as ‘I ate slowly, putting my utensils or food down between bites’ and ‘I checked for feeling of fullness after every bite’. Responses were given on a Likert-type scale of “never”, “sometimes” and “always”, and converted to a scale ranging from 0 to 100 with higher score denoting better adherence. The BSSQ has been validated among bariatric surgery patients and demonstrated good reliability and construct validity [61]. Cronbach alpha was α = .68 for the 2-year post-operative assessment in the present data.

#### Self-efficacy for physical activity

One’s belief in their capability to perform regular physical exercise was assessed with the 10- item Exercise Self-Efficacy Scale (ESES) [62, 63]. Items start with ‘I am confident’ and include, for example, ‘that I can accomplish my physical activity and exercise goals that I set’ and ‘that I can be physically active or exercise even when I am feeling depressed’. Answers are given on a on a four-point scale from 1 *(not at all true*) to 4 (*always true*), generating a total score ranging from 10 to 40, with higher score indicating better self-efficacy. Internal consistency was excellent in the present sample (Cronbach’s alpha = 0.92).

#### Physical activity

The Exercise Behaviour scale was used to describe the time spent on various forms of exercise in the past week [64]. The scale includes six items, each specifying a different type of exercise, such as stretching or strengthening exercises, swimming, or aerobic exercise. Responses are given on a 5-point scale ranging from 0 (*none*) to 4 (*more than 3 h/week*), converted into minutes/week. The six items were summed into a total score indicating the amount of time patients spent exercising, ranging from 0 to 720 min/week.

### **Statistical analysis**

Descriptive statistics were used to analyse patients’ demographic characteristics. A Pearson correlation analysis was conducted to determine the relationship between the continuous study variables and the Spearman rank-order correlation for associations with gender. Continuous variables presented as means and standard deviations and categorical data as frequencies and percentages. Data management and analysis were performed using IBM SPSS Statistics version 27.0 for Windows (2020). Statistical significance was determined with *p* < .05.

To test our primary hypotheses, we conducted four serial mediation analyses using PROCESS version 4.0 [65]. In the first serial mediation model, we predicted the influence of preoperative attachment anxiety on 2-year post-operative diet adherence, as mediated by 1-year post-operative self-esteem and 2-year post-operative self-efficacy to control eating, while controlling for attachment avoidance. The second serial mediation model was identical except the covariate and predictor variables were switched (i.e., avoidance as predictor and anxiety as covariate), to assess the indirect effect between avoidant attachment and diet adherence. In the third and fourth models, the eating-related factors were replaced by exercise-related factors (i.e., self-efficacy to exercise as the second mediator, and physical activity as the outcome variable), but otherwise followed the same configuration as the 1st and 2nd models. To correct for heteroscedasticity in the errors of estimation, we used heteroscedasticity-consistent standard errors (HC3) [66]. To determine whether the indirect effect was significant, we calculated bias-corrected confidence intervals (BC CIs) for the indirect effect by bootstrapping from 10,000 subsamples. If the lower and upper limit confidence intervals do not cross zero, the indirect pathway is deemed significant and mediation present. To achieve a power of 0.8 for mediation analysis using the bias-corrected bootstrapping approach and assuming an effect size of 0.26 for *α*-path and of 0.39 for *β*-path, a sample size of 115 is required, as recommended by Fritz and Mackinnon [67]. The current sample size was therefore deemed sufficiently powered.

## Results

### Study population

One patient was detected as an outlier on study variables and excluded from the final sample. The patients included in the analyses (*n* = 263) were older (mean age 47.73 ± 10.45 years) compared to those who were excluded from the analyses (*n* = 323, *M* = 45.36 ± 11.32 years, *p =* .009). Similarly, excluded patients had higher preoperative BMI than patients included in the analyses (*M* = 39.71 ± 4.42 kg/m^2^, *p* = .028).

**Table 1:** Sociodemographic Characteristics of Participants at Baseline (n = 263)

	*n*	*%*
Gender, female	199	75.7
Age (mean, SD) [range 20.4–65.3 years]	47.73	10.45
**Race**		
Caucasian	235	89.40
South American	10	3.80
Mediterranean	5	1.90
East Asian	4	1.50
Black	3	1.10
Other	3	1.10
West Asian	2	0.80
North African	1	0.40
**Marital status**		
Married/ partnered	196	74.50
Single	47	17.90
Divorced/widowed	19	7.20
Other	1	0.40
**Relationship duration (years)**	14.33	13.62
**Having children, yes (median = 2 children)**	202	76.80
**Highest educational level**		
Lower general education / primary education, or a part of it	4	1.50
General education/ high school	80	30.40
Secondary vocational education	111	42.20
Higher professional education	46	17.50
Scientific education (university)	16	6.10
Otherwise, namely	6	2.30
**Employment**		
Employed (full or part time)	208	79.10
Disabled for work	16	6.10
Homework	15	5.70
Voluntary /unpaid work	10	3.80
Searching for work	8	3.00
Retired	3	1.10
Study	3	1.10
**Start of obesity**		
Childhood	85	32.30
Puberty	64	24.30
Adulthood	107	40.70
Missing	7	2.70
**Type of surgery**		
Roux-en-Y Gastric Bypass	235	89.40
Laparoscopic Omega loop Gastric Bypass	22	8.40
Sleeve gastrectomy	6	2.30

Table 1 shows the demographic characteristics of the sample. The final study sample included 263 patients, who were predominantly female (75.7%), of Caucasian race (89.4%), lived with a partner or were married (74.5%), and had at least 1 child (76.8%). Before the operation, the mean BMI was 38.98 (± 3.61) kg/m^2^ and mean weight 115.02 (± 14.928) kg. Patients lost on average 32.9 kg 2 years after the surgery (*t*(260)= -53.93, *p* < .001), which corresponded to 43.3%AWL and 28.7%TWL. The most common surgical method was laparoscopic- Roux-en-Y Gastric Bypass, performed on 235 patients (89.4%), whereas 22 patients underwent laparoscopic-omega loop- gastric bypass and six patients underwent a sleeve gastrectomy.

Pearson correlation coefficients, means and standard deviations among all the study variables are presented in Table 2. Age, gender or preoperative BMI were not significantly correlated with the study variables and thus were excluded from the models. Mean scores for 2 years post-operative dietary adherence and exercise behaviour scale indicated moderate levels of dietary adherence and low frequency of physical activity within the sample.

**Table 2:** Zero-order correlations and descriptive statistics between study variables

		M	SD		1		2		3		4		5		6		7		8		9		10		11		12	
**1**	Self-esteem 12 m post-op	22.91	5.17	--																								
**2**	Self-efficacy for eating 24 m post-op	74.33	19.19	0.28**		--																						
**3**	Eating self-management 24 m post-op	61.63	18.98	0.03		0.25**		--																				
**4**	Self-efficacy to exercise 24 m post-op	34.70	5.68	0.24**		0.27**		0.10		--																		
**5**	Physical activity 24 m post-op	204.01	155.69	0.07		0.12		0.12		0.34**		--																
**6**	Avoidant attachment style (pre-op)	20.91	8.65	− 0.39**		− 0.10		0.04		− 0.05		− 0.04		--														
**7**	Anxious attachment style (pre-op)	23.19	10.88	− 0.49**		− 0.25**		0.06		− 0.11		0.06		0.47**		--												
**8**	Age (pre-op)	47.73	10.45	0.10		0.001		− 0.04		− 0.07		0.06		0.07		− 0.03		--										
**9**	Gender	0.76	0.43	− 0.13*		− 0.07		0		0.04		0.02		− 0.14*		0.01		− 0.21**		--								
**10**	BMI pre-op	38.98	3.60	0.07		0.05		0.10		0.06		0.11		0.03		− 0.03		− 0.15*		− 0.01		--						
**11**	BMI 12 m post-op	27.51	3.50	0.12*		0.04		0.01		− 0.01		0.07		− 0.06		− 0.14*		0.11		− 0.16**		0.67**		--				
**12**	BMI 24 m post-op	27.75	3.89	0.14*		− 0.02		− 0.02		− 0.02		0.08		− 0.10		− 0.12*		0.06		− 0.20**		0.61**		0.92**		--		

*Notes.*

Statistically significant correlations are marked with * = *p* < .05. ** =*p* < .01.

*N* = 263, except for Eating self-efficacy and BMI at 24 m post-operation (*n* = 261).

Spearman’s Rank-Order Correlation reported for Gender.

### Mediation analyses

#### Attachment anxiety, avoidance and Dietary Adherence

The results of the first and second serial mediation analyses are presented in Table 3 (and in Additional file Figure A2 and Figure A3). Preoperative attachment anxiety and avoidance predicted poorer post-operative self-esteem (*p* < .001) 1 year after bariatric surgery and explained 28% of the variance in self-esteem. Higher self-esteem, in turn, significantly predicted better self-efficacy for eating (*d*_*21*_ = 0.80, *p* = .003), which further predicted better diet adherence (*b*_*2*_ = 0.28, *p* < .001). Attachment anxiety predicted lower self-efficacy to control eating behaviours significantly (*a*_*2*_*=* -0.33, *p* = .010) whereas attachment avoidance did not (*C*_*2*_ *=* 0.15, *p* = .301). When the mediators were not included in the model, neither attachment anxiety nor attachment avoidance significantly predicted post-operative diet adherence (total effects for anxiety *c* = 0.09, *t*(258) = 0.81, *p* = .417, and for avoidance *C*_*3*_ = 0.03, *t*(258) = 0.19, *p =* .853). Attachment anxiety and avoidance alone accounted for 0.9% of the variance in post-operative dietary adherence. When attachment anxiety and avoidance and both mediators were included in the model, they explained 8.1% of the variance in dietary adherence, and the model was significant (*F*(4, 256) = 5.83, *p* < .001).

#### Direct and indirect effects

When both mediators were included in the model, the direct effect between attachment anxiety and diet adherence became significant (*c’*= 0.24, *t*(256) = 2.04, *p* = .043), and significant indirect relationships through self-efficacy to control eating (*95% BC CI*: -0.19, -0.03), and through self-esteem and self-efficacy to control eating (*IE*= -0.04, *SE* = 0.02, *95% BC CI*: -0.09, -0.02) were evident. The total indirect effect of attachment anxiety to dietary adherence through the two mediators was significant with a coefficient of -0.15 and 95% bias-corrected confidence interval excluding zero (-0.29 to -0.05). The indirect effects are presented in Table 5.

Conversely, the direct relationship between attachment avoidance and diet adherence was not significant when the mediators included in the model (*C’*_*3*_= 0.02, *t*(256) = 0.15, *p* = .877). Further, only the serial mediation between attachment avoidance and diet adherence through self-esteem and self-efficacy was significant (*IE*= -0.03, *SE* = 0.01, *95% BC CI*: -0.07, -0.01). Accordingly, the total indirect effect was nonsignificant with the bias-corrected 95% confidence interval ranging from − 0.11 to 0.12.

**Table 3:** Unstandardized Regression Coefficients, Standard Errors (with HC3 correction), t- and p-values, and Model Summary for Serial Multiple Mediator Model for eating behaviours, depicted in Fig. 1

	Consequent														
	M1 (self-esteem)					M2 (SE eating 24 m)					Y (Diet adherence 24 m)				
Antecedent		Coeff.	*SE*	*t*	*p*		Coeff.	*SE*	*t*	*p*		Coeff.	*SE*	*t*	*p*
X (AS anxiety)	*a*_*1*_	-0.19	0.03	-6.25	0.000	*a*_*2*_	-0.33	0.13	-2.58	0.010	*c’*	0.24	0.12	2.04	0.043
M1 (Self-esteem 12 m)						*d*_*21*_	0.80	0.27	2.99	0.003	*b1*	0.09	0.25	0.38	0.701
M2 (SE eating 24 m)											*b2*	0.28	0.06	4.55	0.000
Cov (AS avoidance)	*C*_*1*_	-0.12	0.04	-2.83	0.005	*C*_*2*_	0.15	0.15	1.04	0.301	* C’*_*3*_	0.02	0.15	0.16	0.877
CONSTANT	*i*_*M1*_	29.90	0.83	36.12	0.000	*i*_*M2*_	60.38	8.44	7.15	0.000	*i*_*Y*_	32.70	8.98	3.64	0.000
		*R*^*2*^ = 0.28					*R*^*2*^ = 0.10					*R*^*2*^ = 0.08			
		* F*(2, 258) = 39.19, *p* = .000					* F*(3, 257) = 11.30, *p* = .000					* F*(4, 256) = 5.83, *p* = .000			

*Notes. n* = 261.

**Table 4:** Unstandardized Regression Coefficients, Standard Errors (with HC3 correction), t- and p-values, and Model Summary for Serial Multiple Mediator Model for exercise behaviours, depicted in Fig. 1

	Consequent														
	M1 (self-esteem 12 m)					M2 (SE exercise 24 m)					Y (Physical activity 24 m)				
**Antecedent**		Coeff.	SE	*t*	*p*		Coeff.	SE	*t*	*p*		Coeff.	SE	*t*	*p*
X (AS anxiety)	*a*_*1*_	-0.19	0.03	-6.17	0.000	*a*_*2*_	-0.00	0.04	-0.05	0.963	*c’*	2.09	1.05	1.99	0.048
M1 (Self-esteem 12 m)						*d*_*21*_	0.28	0.07	4.12	0.000	*b1*	0.77	2.11	0.36	0.716
M2 (SE exercise 24 m)											*b2*	9.56	1.41	6.80	0.000
Cov (AS avoidance)	*C*_*1*_	-0.12	0.04	-2.82	0.005	*C*_*2*_	0.03	0.04	0.78	0.434	* C’*_*3*_	-1.45	1.16	-1.25	0.213
CONSTANT	*i*_*M1*_	29.79	0.83	36.10	0.000	*i*_*M2*_	27.58	2.32	11.86	0.000	*i*_*Y*_	-163.42	83.65	-1.95	0.052
		*R*^*2*^ = 0.27					*R*^*2*^ = 0.06					*R*^*2*^ = 0.13			
		* F*(2, 260) = 38.42, *p* = .000					* F*(3, 259) = 7.75, *p* = .001					* F*(4, 258) = 12.41, *p* = .000			

#### Attachment anxiety, avoidance and physical activity

The serial mediation models between preoperative attachment anxiety (and attachment avoidance as a covariate) and 2 years post-operative physical activity through self-esteem and self-efficacy for exercise behaviours are presented in Table 4. Neither attachment anxiety nor avoidance predicted self-efficacy to be physically active (*a*_*2*_*=* -0.00 and *C*_*2*_ = 0.03, respectively, *p*s > .05) two years after the surgery. When the mediators were not included in the model, attachment anxiety (*c* = 1.42, *p* = .149) and avoidance (*C*_*3*_*=* -1.54 *(p* = .188) did not predict physical activity two years after the surgery (total effects). Without self-esteem and self-efficacy to be physically active in the model, attachment anxiety and avoidance explained 0.9% of variance in post-operative physical activity. When attachment anxiety and avoidance and both mediators were included in the model, they explained 13.3% of the variance in physical activity, and the model was significant (*F*(4, 258) = 12.41, *p* < .001).

#### Direct and indirect effects

The direct relationship between attachment anxiety and physical activity was significant when mediators were included in the model (*c’*= 2.09, *SE* = 1.05, *p* = .048). As hypothesized, attachment anxiety had an indirect effect on physical activity through self-esteem and self-efficacy to exercise (*95% BC CI*: -0.89, -0.26), while the indirect effects via each mediator separately were not significant (i.e., serial mediation only).

Attachment avoidance did not predict 2-year post-operative physical activity when the mediators were included in the model (*C’*_*3*_*=* -1.45, *t*(258)= -1.25, *p* = .213). Similarly, only the serial mediation between attachment avoidance and 2- year post-operative physical activity through self-esteem and self-efficacy to exercise was significant (*95% BC CI*: -0.68, -0.12).

**Table 5:** Indirect effects (IE) and bootstrapped standard errors (SE) for the serial mediation models

Path	IE	SE	95% CI
**Diet adherence**			
AS anxiety- Self-esteem- Diet adherence (cov: avoidance)	-0.02	0. 05	-0.12; 0.07
AS anxiety- Self-efficacy- Diet adherence	-0.09	0.04	-0.19; -0.03
AS anxiety - Self-esteem- Self-efficacy- Diet adherence	-0.04	0.02	-0.09; -0.02
Total (anxiety- Diet adherence)	-0.15	0.06	-0.29; -0.05
AS avoidance- Self-esteem- Diet adherence (cov: anxiety)	-0.01	0.03	-0.09; 0.04
AS avoidance- Self-efficacy- Diet adherence	0.04	0.05	-0.03; 0.14
AS avoidance - Self-esteem- Self-efficacy- Diet adherence	-0.03	0.01	-0.07; -0.01
Total (avoidance- Diet adherence)	0.00	0.06	-0.11; 0.12
**Physical activity**			
AS anxiety- Self-esteem- Exercise behaviour (cov: avoidance)	-0.15	0. 40	-0.97; 0.62
AS anxiety- Self-efficacy- Exercise	-0.02	0.34	-0.71; 0.62
AS anxiety - Self-esteem- Self-efficacy- Exercise	-0.51	0.16	-0.89; -0.26
Total (anxiety- Exercise)	-0.67	0.50	-1.68; 0.28
AS avoidance- Self-esteem- Exercise behaviour (cov: anxiety)	-0.0 9	0.27	-0.75; 0.37
AS avoidance- Self-efficacy- Exercise	0.32	0.41	-0.48; 1.15
AS avoidance - Self-esteem- Self-efficacy- Exercise	-0.33	0.14	-0.68; -0.12
Total (avoidance- Exercise)	-0.00	0.03	-0.06; 0.05

In other words, preoperative attachment anxiety predicted lower 1- year post-operative self-esteem (*a*_*1*_= -0.19, *p* < .001), as did preoperative attachment avoidance (*c*_*1*_= -0.12, *p* = .005). Higher self-esteem predicted better self-efficacy to be physically active two years after operation (*d*_*21*_ *=* 0.28, *p* < .001), which, in turn, predicted more frequent physical activity (*b*_*2*_ = 9.56, *p* < .001).

## Discussion

The current study aimed to examine the mediational role of self-esteem and health self-efficacy between attachment anxiety and avoidance and health behaviours (i.e., diet adherence and physical activity). This work could generate fresh insights into the mechanism between attachment and health behaviours, and how patients could be supported to follow a healthy lifestyle. We investigated this mechanism among bariatric surgery patients with a longitudinal dynamic model, allowing us to examine the relations between the study variables over time and obtain indications for the sequence of events. One of the more significant findings to emerge from this study is that post-operative self-esteem and health self-efficacy mediated the relationships between preoperative attachment anxiety and avoidance and 2-year post-operative diet adherence and physical activity. Yet, the full models explained only a small part of the variation in the outcome variables (8% of dietary adherence and 13% of physical activity).

Interestingly, higher attachment anxiety was associated with better dietary adherence and more exercise behaviours when the mediators were held constant (positive direct effects), while the indirect effects were negative. Thus, patients equal on post-operative self-esteem and self-efficacy for eating and exercise behaviours but who were one unit higher on preoperative attachment anxiety were estimated to have 0.24 units better dietary adherence and 2.09 units more physical activity two years after bariatric surgery than patients scoring one unit less on preoperative attachment anxiety. In other words, patients equal on post-operative self-esteem and self-efficacy, but who were more anxiously attached preoperatively were estimated to have slightly better post-operative dietary adherence and to be more physically active. Even though the changes in the units are small, these results imply that greater attachment anxiety is associated with better diet and exercise behaviours for patients with similar self-esteem and health self-efficacy. The negative indirect effects indicate that higher attachment anxiety is associated with poorer dietary adherence and less physical activity through poorer self-esteem and eating self-efficacy. A possible explanation for these results could be that patients with similar self-esteem and self-efficacy level, yet more anxiously attached, can to turn their hyper-activated attachment system into their advantage. Perhaps these individuals are more self-compassionate or conscientious, and have an *advantageous attachment anxiety*, manifesting as vigilance for their health and diligence to their lifestyle regimen. Maybe a similar mechanism is in place with individuals described as *healthy neurotic*, whose high neuroticism leads them to worry over their health compulsively, but due to their high conscientiousness can act upon it, ultimately benefitting their health [68, 69].

### Attachment, self-esteem, and health self-efficacy

Another important finding was that higher preoperative attachment anxiety and avoidance were associated with poorer post-operative self-esteem, which is in line with previous findings [e.g., 36–39]. Further, better self-esteem was associated with higher belief in own competence to control both eating and exercise behaviours. A previous study has even demonstrated that self-esteem and self-efficacy have a reciprocal relationship [70]. High self-esteem seems to boost individuals’ belief in their competences and encourages them to behave accordingly. Thus, patients’ beliefs about themselves are highly important for their health behaviours, especially among patients with high attachment anxiety and avoidance. Lastly, attachment anxiety was associated with poorer self-efficacy for controlling eating behaviour, but not exercising, in the present sample. Meanwhile, the relationships between attachment avoidance and self-efficacy to control eating and exercise behaviours were not significant.

Although no other study has investigated the relation between attachment and health behaviours through self-esteem and self-efficacy, the present results reflect those of previous studies. Patients in the current sample scored relatively low on both attachment anxiety and avoidance, similar to the results of other studies [e.g., 19, 24,71]. Self-esteem has also been found to mediate attachment style and health behaviours among students [72]. Our study extends these findings using a dynamic, longitudinal model allowing an in-depth examination of these variables over time.

### Post-operative health behaviours

Regarding health behaviours, patients in the current sample reported adherence to dietary recommendations two years after the surgery, which was on the upper half of the scale range. Meanwhile, the amount of time spent exercising was low. Patients are forced to eat small portions, and reduced fat and carbohydrate intake by the anatomic changes after surgery and have a higher chance of dumping or abdominal pain in case of non-adherence. Another explanation may be that patients focus more on changing their eating behaviours after bariatric surgery as poor eating behaviours are commonly blamed for being overweight (both subconsciously and societally), reflected by the vast number of various dieting programs, books and articles promising easy weight loss with the newest diets. Therefore, this diet-trance may overshadow the importance and benefit of regular physical activity for weight loss. Based on our finding of limited frequency of physical activity, patients should be encouraged to exercise more regularly and given advice on appropriate physical activity during the surgical screening. It must be noted that part of the follow-up data was collected during the Covid-19 pandemic, during which access to sport facilities was largely limited. Therefore, these findings must be interpreted cautiously and repeated with other samples and longer follow-up data.

### Strength and limits

The current study benefitted from the use of longitudinal data, which allowed the rigorous testing of a dynamic mediational model and obtained new insights into the mechanism between attachment anxiety, avoidance and health behaviours after bariatric surgery. Previous studies have relied on cross-sectional samples, thus, our results provide much-needed insight into associations over time. The majority of prior research on bariatric surgery patients has focused on eating-related factors. Therefore, studies focusing on other health behaviours important for weight loss and management are needed. Secondly, patients were assessed after they had been approved for bariatric surgery, thus reducing the urge to ‘socially enhance’ their responses, rendering the data more reliable. Lastly, the assessments were mainly conducted during hospital visits, ensuring little missing data.

A limitation of the study was the reliance on self-report measures, which are subject to bias [73]. Previous studies have recognised that avoidantly attached individuals tend to underreport their general distress and bodily symptoms [74, 75]. Therefore, replication using a clinician-administered assessment, such as Adult Attachment Interview [76], should be considered to obtain a more direct assessment of attachment style. Although the measures used in the study have previously been validated, more objective measures of eating and exercise behaviours would have been desirable, such as food diaries and movement trackers for collecting more specific data. Additionally, this study did not evaluate post-operative smoking or alcohol use, which are important health behaviours impacting overall health. Future studies could include these behaviours as well as the presence of clinical conditions, such as osteoarthritis and cardiovascular disease, in the models to obtain a more holistic image of the patients’ health after bariatric surgery. Finally, the generalizability of our findings is restricted to bariatric surgery patients who were predominately Caucasian women.

### Conclusions and clinical implications

Taken together, the findings from this investigation suggest that while individuals with high pre-operative attachment anxiety are expected to have lower post-operative self-esteem and poorer self-efficacy for health behaviours, high self-esteem can support patients’ ability to control their eating and exercise behaviours, resulting in better diet adherence and more physical activity two years after bariatric surgery. The individual pathways between preoperative attachment avoidance, post-operative self-esteem, health self-efficacy and health behaviours were not significant. However, the relationships between preoperative attachment avoidance and 2-year post-operative diet adherence and exercise behaviours were significantly mediated through self-esteem and health self-efficacy.

The findings reported here shed new light on the role of adult attachment in health behaviours and the potential of self-esteem in promoting health self-efficacy among bariatric surgery patients. Our results suggest that helping patients feel more worthy and reinforcing their beliefs about their own competences could lead to higher engagement with healthy lifestyle, better weight management skills, and adherence to treatment protocols, ultimately helping patients to achieve their bariatric surgery goals. It might be beneficial to consider patients’ attachment during the presurgical assessment and offer additional treatment opportunities, such as Group Psychodynamic Interpersonal Psychotherapy or ‘security- priming’ [77, 78], to foster more secure attachments. Moreover, self-efficacy could be enhanced by helping individuals become more aware of specific situations in which efficacy may be low, practicing desired behaviours in these situations, and promoting personal coping skills [79]. Especially when experiencing lapses in desired health behaviours, low self-efficacy may lead a person to attribute the lapse to personal weakness and reduce their chances of recovering from such an event, possibly leading to sustaining unhealthy habits [80]. As high attachment anxiety and avoidance and poor self-esteem and self-efficacy could persist after surgery, especially among patients with poor results, prospective studies are needed to investigate the effectiveness of interventions targeting attachment style, self-esteem and self-efficacy pre- and post-surgery as well as the long-term effects of post-operative health competences and differences in attachment style on health behaviours and health outcomes (e.g., weight loss, quality of life and body image satisfaction) after bariatric surgery. Although we did not test it in the current study, higher self-esteem could also predict better psychological outcomes after bariatric surgery, such as better body image satisfaction, as these are closely related to self-worth.

### What is already known on this subject?

Attachment style can impact health by influencing how we respond to stress, regulate emotions, adhere to treatments, and perceive and report symptoms. Patients with severe obesity undergoing bariatric surgery have often been reported to have high attachment anxiety and avoidance. These characteristics are also associated with poor eating behaviours and weight maintenance.

### What this study adds?

This longitudinal study investigated the serial mediation between preoperative attachment and 2-years post-operative health behaviour through 1-year post-operative self-esteem and health self-efficacy. The relationships between preoperative attachment anxiety and avoidance and diet adherence and physical activity two years after bariatric surgery are mediated by post-operative self-esteem and self-efficacy for eating and exercise behaviours.

The study contributes to our understanding of the mechanism between attachment and health behaviours, and suggests a potential pathway through self-esteem and self-efficacy. Promoting post-operative self-esteem may support patients’ ability to control their eating and exercise behaviours, which could be important for enhancing post-operative diet adherence and physical activity, especially among patients with high attachment anxiety or avoidance.

### Electronic supplementary material

Below is the link to the electronic supplementary material.

Supplementary Material 1

## Data Availability

Deidentified study data will be shared upon reasonable request by contacting the corresponding author. The full study protocol is published and available to the public [55].
