# Supplementary material for: Attachment style and post-bariatric surgery health behaviours: the mediating role of self-esteem and health self-efficacy
Source: BMC Psychol. 2023 Aug 25;11:248. doi: 10.1186/s40359-023-01273-5 (PMC10464092; doi:10.1186/s40359-023-01273-5)
Supplement: Supplementary file 1 — Supplementary Material 1 [file 40359_2023_1273_MOESM1_ESM.docx]

**Additional files**

**
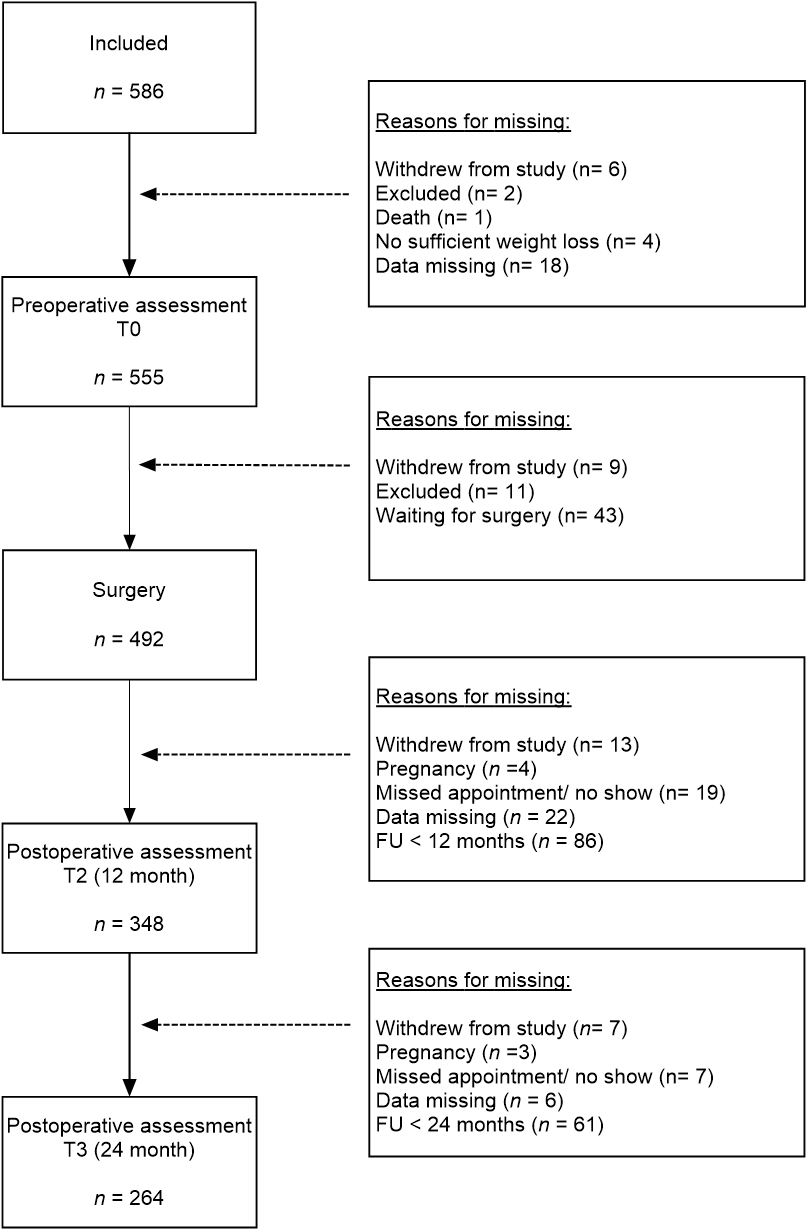
Figure A1.** *Flowchart of data inclusion.*

**Figure A2.**

*
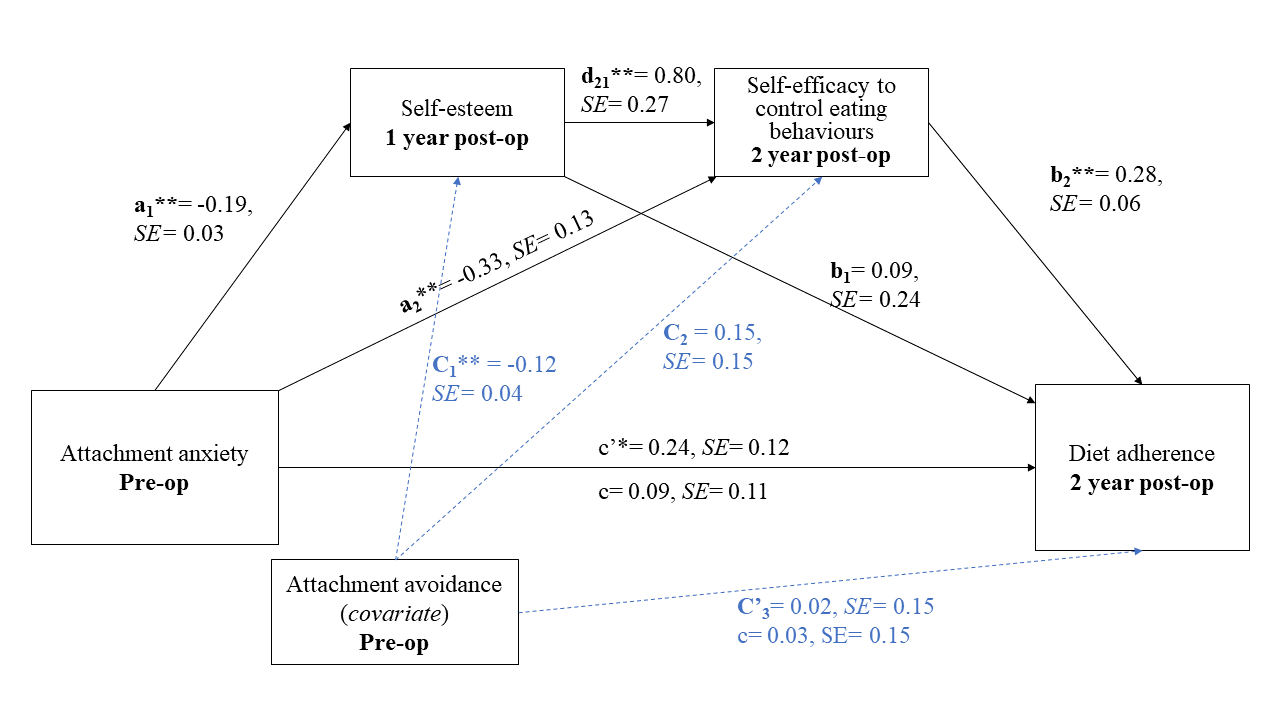
Serial mediation model. Unstandardized regression coefficients and standard errors (with HC3 correction) are presented. *= p < .05, **= p < .01*

**Figure A3.**

***
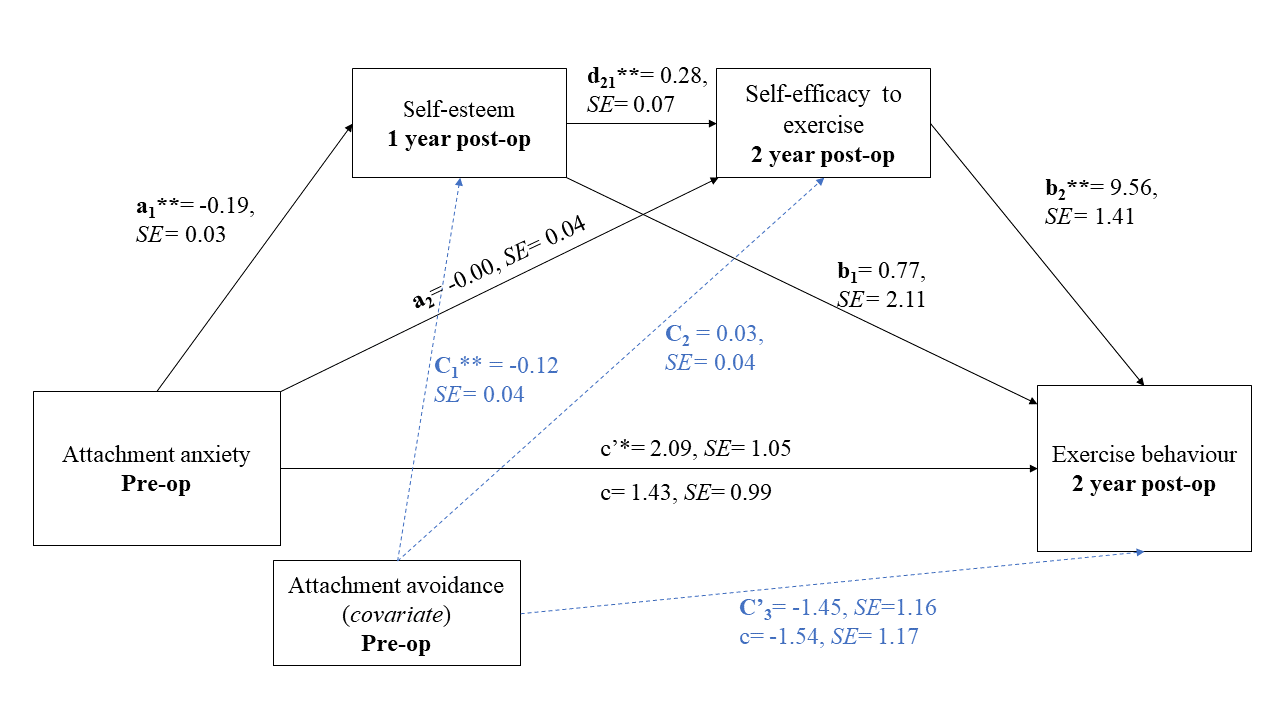
***Serial mediation model. Unstandardized regression coefficients and standard errors
